# Screening and Identification of an H-2K^b^-Restricted CTL Epitope within the Glycoprotein of Hantaan Virus

**DOI:** 10.3389/fcimb.2016.00151

**Published:** 2016-11-25

**Authors:** Rui-xue Ma, Lin-feng Cheng, Qi-kang Ying, Rong-rong Liu, Tie-jun Ma, Xiao-xiao Zhang, Zi-yu Liu, Liang Zhang, Wei Ye, Fang-lin Zhang, Zhi-kai Xu, Fang Wang, Xing-an Wu

**Affiliations:** Department of Microbiology, School of Basic Medicine, Fourth Military Medical UniversityXi'an, China

**Keywords:** HTNV, epitope, glycoprotein, IFN-γ, infection

## Abstract

The cytotoxic T lymphocyte (CTL) response plays a key role in controlling viral infection, but only a few epitopes within the HTNV glycoprotein (GP) that are recognized by CTLs have been reported. In this study, we identified one murine HTNV GP-derived H2-K^b^-restricted CTL epitope in C57BL/6 mice, which could be used to design preclinical studies of vaccines for HTNV infection. First, 15 8-mer peptides were selected from the HTNV GP amino acid sequence based on a percentile rank of <=1% by IEDB which is the most comprehensive collection of epitope prediction and analysis tool. A lower percentile rank indicates higher affinity and higher immune response. In the case of the consensus method, we also evaluated the binding score of peptide-binding affinity by the BIMAS software to confirm that all peptides were able to bind H2-K^b^. Second, one novel GP-derived CTL epitope, GP6 aa456-aa463 (ITSLFSLL), was identified in the splenocytes of HTNV-infected mice using the IFN-γ ELISPOT assay. Third, a single peptide vaccine was administered to C57BL/6 mice to evaluate the immunogenic potential of the identified peptides. ELISPOT and cell-mediated cytotoxicity assays showed that this peptide vaccine induced a strong IFN-γ response and potent cytotoxicity in immunized mice. Last, we demonstrated that the peptide-vaccinated mice had partial protection from challenge with HTNV. In conclusion, we identified an H2-K^b^-restricted CTL epitope with involvement in the host immune response to HTNV infection.

## Introduction

Hantaan virus (HTNV), the prototype of the Hantavirus genus, is a rodent-borne pathogen that is the major causative agent of hemorrhagic fever with renal syndrome (HFRS; Abbott et al., 1999). The genome of HTNV consists of L, M, and S segments, which encode the viral RNA-dependent RNA polymerase, glycoprotein, and nucleoprotein, respectively (Cheng et al., 2014). HFRS is a severe, life-threatening illness characterized by fever, hemorrhage, and renal failure and is a serious public health threat in China (Hansen et al., 2015). A total of 136,012 cases were reported during 2005–2015 in mainland China, with a case-fatality rate as high as 10–15% (Zhang et al., 2010; Kang et al., 2012). To date, several types of inactivated vaccines targeting the pathogens that cause HFRS have been licensed (Liu et al., 2016). However, these inactivated vaccines have many shortcomings, including poor immunogenicity, which hinders the production of neutralizing antibodies or cell-mediated immunity (Song et al., 1992), and the concern of safety when using inactivated vaccines, which may contain some infectious particles. Furthermore, there is no study reporting that it could establish long-term memory immunity (Jiang et al., 2015). Thus, it is necessary to develop an alternative immunotherapy strategy that can induce robust humoral immune response to produce therapeutically effective antiviral antibodies and T cell-mediated autoimmune responses. However, epitope based vaccines represent a powerful approach to induce a specific immune response against the selected epitopes, avoiding the side effects of other unfavorable epitopes in the complete antigen. What's more, epitope based vaccine has other considerable advantages, including increased safety, the opportunity to engineer the epitopes rationally for increased potency and breadth, and the ability to focus the immune response on conserved epitopes CTL epitope-specific T cell immunity demonstrate to be a long-term approach immune strategy. Moreover, the epitope based vaccine approach has been shown to be successful in various infectious diseases, such as Neisseria meningitides infection, HIV (Park et al., 2016), RSV, and tuberculosis (Paul et al., 2013). Therefore, a potent T cell-activating peptide vaccine based on the HTNV structural protein may be a promising method of disease control (Ma et al., 2013).

The antigenicity of Hantaviruses largely depends on two structural proteins, the nucleocapsid protein (NP) and envelope glycoprotein (GP) (Zeier et al., 2005). NP is highly immunogenic, inducing vigorous cellular and humoral immune responses in humans, and conserved among viral species. GP is not only responsible for receptor binding and membrane fusion, but is also considered the main target of neutralizing antibodies (Wang et al., 2011). T cells are essential components of the immune response to most viral infections. Therefore, epitopes of HTNV NP that elicit CD4^+^ and CD8^+^ cytotoxic T lymphocyte responses have been extensively studied (Ennis et al., 1997; Van Epps et al., 1999; Lee et al., 2002). Viral GP-specific T cell responses have also been discovered (Ma et al., 2015). Protective immunity elicited through infection with recombinant HTNV-glycoprotein in murine models has also been demonstrated (Yu et al., 2013). Recent interest has emerged in the concept of HTNV glycoprotein serving as a potent immunogen to induce T cell responses (Manigold et al., 2010). However, T cell epitopes on the HTNV glycoprotein have not been fully identified, and specific responses to these immunogens remain largely unknown.

During viral infection, CTLs are important for viral clearance and preventing immunopathology in humans (Kiepiela et al., 2007). To create a mouse model for studying the role of HTNV-specific T cells *in vivo*, we employed a newly identified mouse CTL epitope on the HTNV glycoprotein. In our study, we describe a CTL epitope on HTNV GP and analyze the cellular immune response against HTNV. Our findings could support the production of an effective peptide vaccine for HTNV infection in humans. To our knowledge, this is the first report about mouse CTL epitopes on the glycoprotein of HTNV.

## Materials and methods

### Animals

Pathogen-free C57BL/6 female mice between 8 and 10 weeks old were purchased from the animal research center of the Fourth Military Medical University (Xi'an, China). The mice were cared for in accordance with the Guide for the Care and Use of Laboratory Animals. The animals were acclimated to the laboratory environment for 5–7 d before use. While in their home cage environment, the animals were allowed free access to a standard animal diet and tap water. The room was maintained at 20–23°C with a 12 h/12 h light/dark cycle. The animals were deeply anesthetized using inhaled isoflurane (1–3% or as needed) before all operations. All efforts were made to minimize animal suffering, to reduce the number of animals used, and to utilize alternatives to *in vivo* experiments whenever appropriate or feasible. The animal protocol was approved by the Fourth Military Medical University Medical Ethics Committee (Xi'an, Shaanxi, China; approval no.XJYYLL-2015510). Experiments were performed in age-matched groups.

### Viruses and cells

HTNV strain 76–118 was obtained from our repository. EL-4 and P815 cells, which express H-2^b^ and H-2^d^ as MHC class I molecules, were obtained from American Type Culture Collection (ATCC; Manassas, VA, USA). EL-4 cells were cultured in Dulbecco's Modified Eagle's Medium (DMEM; Invitrogen, Carlsbad, CA, USA) supplemented with 10% horse serum (HyClone, Logan, UT, USA). All other cells were maintained in DMEM that was supplemented with 10% fetal bovine serum (FBS) (HyClone, Logan, UT, USA).

### Synthesis of peptides

The 15 8-mer peptides used in the ELISPOT screens correspond to the glycoprotein of HTNV strain 76–118 (Table 1). All of these peptides were synthesized with greater than 98% purity as determined by high-performance liquid chromatography (HPLC) and resuspended in sterile phosphate-buffered saline (PBS) solution containing DMSO (1 mg/mL). Each peptide is H-2K^b^-restricted. HTNV nucleoprotein (NP) aa221–aa228 (SVIGFLAL) derived from the HTNV nucleoprotein was used as a positive control epitope (Park et al., 2000). We used computer algorithms in Immune Epitope Database and Analysis Resources (IEDB-http://tools.iedb.org/main/) to identify optimal peptide epitopes within the reactive 8-mer peptides.

**Table 1:** **Synthetic peptides on the glycoprotein of HTNV predicted to bind with H2-K^**b**^**.

**Serial number of peptide**	**8-mer peptide**	**Octamer sequence^a^**	**Percentile_rank^b^**	**Binding score^c^**
GP 1	39~46:	SVIGYVEL	0.75	7.2
GP 2	44~51	VELPPVPL	0.75	4.5
GP 3	64~71	SMDNHQSL	0.75	7.4
GP 4	208~215	IVCFFVAV	0.55	3.7
GP 5	420~427	VNFVCQRV	0.5	5.7
GP 6	456~463	ITSLFSLL	0.25	10.3
GP 7	489~496	VTFCFGWV	0.2	6.8
GP 8	499~506	PAITFIIL	0.5	8.1
GP 9	797~804	ITIRYSRR	0.4	4.9
GP 10	845~852	TLLFFGPL	0.4	8.4
GP 11	925~932	QSFNTSTM	0.4	3.4
GP 12	987~994	VGFTLTCL	0.5	4.3
GP 13	1102~1109	FSGNWIVL	0.7	3.6
GP 14	1113~1120	CVFLLFSL	0.85	4.9
GP 15	1114~1121	VFLLFSLV	0.25	3.1
NP 1	221–228	SVIGFLAL	0.2	11.0

a *Corresponding to HTNV (76–118) GP*.

b *A percentile rank in H-2K^b^ restriction, was calculated by IEDB*.

c *The binding score in H-2K^b^ restriction, expressed as arbitrary units, was calculated by the BIMAS software*.

### *Ex vivo* IFN-γ enzyme-linked immunospot (ELISPOT) assay

Identification of the HTNV GP-specific CTL epitopes was performed using the IFN-γ ELISPOT assay (Mabtech, Büro Deutschland, Germany) according to the manufacturer's instructions. After 7 days of infection with HTNV, mice were sacrificed and their spleens harvested. Briefly, splenocytes were prepared and assayed for their ability to secrete IFN-γ during *in vitro* restimulation with antigenic peptides. The positive peptides in the first round screen were subsequently characterized in CD4- or CD8-depleted splenocytes using anti-CD4-coated Dynalbeads (Invitrogen Dynal AS, Oslo, Norway) and anti-CD8-coated Dynalbeads (Invitrogen Dynal AS, Oslo, Norway). Total splenocytes or the isolated T cells were seeded in ELISPOT plates at 5 × 10^5^ cells/well and stimulated with 15 8-mer peptides at a final concentration of 10 μg/mL. Cells with nonspecific phytohemagglutinin stimulation (PHA, 10 μg/mL, Sigma-Aldrich, St. Louis, MO) or without peptide stimulation served as positive and negative controls, respectively. Additionally, spleen cells pulsed with the immunodominant NP221–228 peptide (Park et al., 2000) was used as an epitope positive control. For quantification of *ex vivo* responses, the assay was performed in duplicate. An automated ELISPOT reader (Cellular Technology Limited, USA) was used to count the spots. Spot-forming cells (SFC) were adjusted by subtracting average negative values and expressed as SFC/10^6^ splenocytes. A positive response was defined as having at least 100 SFC/10^6^ input cells. The SFC/10^6^ splenocytes in unstimulated control wells never exceeded 5 spots per well.

### Cell-mediated cytotoxicity assay

The lactate dehydrogenase (LDH)-releasing CytoTox 96 nonradioactive cytotoxicity assay (Promega, Madison, WI, USA) was performed to detect the level of specific cytotoxicity, as described in the manufacturer's protocol. CD8+ T cells isolated with anti-CD8-coated Dynalbeads from the splenocytes of HTNV-immunized mice were used as effector cells. Target cells used in this study were peptide-pulsed EL-4 cells, HTNV-inoculated macrophages, and control cells (P815). Target cells were plated at 1 × 10^4^ cells per well in a volume of 50 μL in wells of a 96-well U-bottomed microtiter plate. The splenocytes (effector cells) were added to a final volume of 50 μL at effector/target (E/T) ratios of 100:1, 50:1, 20:1, and 10:1. The assay plate included the following controls: spontaneous lactate dehydrogenase (LDH) release from effector cells alone (50 μL of effector cells and 50 μL of 10% FBS RPMI-1640 medium), spontaneous LDH release from target cells alone (50 μL of target cells and 50 μL of 10% FBS RPMI-1640 medium), maximum LDH release from target cells alone (50 μL of target cells, 50 μL of 10% FBS RPMI-1640 medium and 10 μL of the lysis solution), a volume correction control (100 μL of 10% FBS RPMI-1640 medium and 10 μL of the lysis solution), and a culture medium background control (100 μL of 10% FBS RPMI-1640 medium). The percentage of cells lysis was calculated according to the following formula: % cytotoxicity = [(*E* − *S*_*t*_ − *S*_*e*_)/(*M* − *S*_*t*_)] × 100% (E, LDH release from effector-target co-culture cells; St, spontaneous LDH release from target cells; Se, spontaneous LDH release from effector cells; M, maximum LDH release from target cells). All of the CTL assays were performed in triplicate and repeated at least twice.

### Intracellular cytokine staining

Two million freshly isolated splenocytes from HTNV-infected mice were stimulated for 4 h with HTNV GP-specific peptides (10 μg/mL for each peptide). Cells stimulated with PHA (0.1 μg/ml, Sigma-Aldrich, St. Louis, MO) or medium alone were used as positive and negative controls, respectively. After washing, splenocytes were stained with antibodies against surface markers, CD3-FITC and CD8-PerCP/Cy5.5 (BD Pharmingen), in different combinations for 40 min at 4°C, followed by washing, fixation and permeabilization with a buffer (BD Biosciences, San Jose, CA). The cells were subsequently stained with antibodies against intracellular markers IFN-γ-PE (BD Pharmingen), for 40 min at 4°C, followed by washing, and acquisition using a FACS Calibur flow cytometer (Becton Dickinson). A total of 100,000 events per sample from the lymphocyte gate were collected for each analysis.

### *Ex vivo* proliferation assay

The CFSE-labeled proliferation assay was performed as previously described. Briefly, 1 × 10^7^/ml PBMCs were labeled with 10 μM 5,6-carboxyfluorescein succinimidyl ester (CFSE, Molecular Probes, OR) at 37°C for 15 min. Labeling was terminated upon the addition of fetal bovine serum and the cells were stimulated with HTNV GP-specific peptides (10 μg/mL). Anti-mouse CD3 (Biolegend) stimulation of PBMCs and no peptide stimulation served as positive and negative controls, respectively. After 2 days, 10% exogenous IL-2 was added. After 5 days, the cells were harvested to detect their expansion capacity. Approximately 100,000 cells were acquired using a FACS Calibur (BD Immunocytometry Systems, California).

### Immunizations

As shown in **Figure 5A**, 8-week-old C57BL/6 (H-2^b^) mice (10 mice/group) were subcutaneously immunized four times with mixed peptide vaccines at 10 day intervals. The mixed peptide vaccine per mouse consisted of 100 μL (100 μg) of GP6 packaged with either 100 μL complete Freund's adjuvant (CFA) at primary immunization or incomplete Freund's adjuvant (IFA) at boost immunization. Each vaccine solution was emulsified. Four control groups were used: the first two groups were immunized with 100 μL (100 μg) of NP 1 packaged with 100 μL Freud's adjuvant or 200 μL (100 μg) of commercially purchased inactivated Hantavirus vaccine (Tianyuan, Hangzhou, Zhejiang, China) per mouse; and the other two groups were given either 200 μL of PBS or 200 μL of Freud's adjuvant per mouse. At 10 d after the last immunization, 5 mice of each group were sacrificed and their splenocytes were isolated for subsequent assays.

### Animal challenge/protection assay

At 10 d after the final boost immunization, the remaining mice of each group received an infectious dose (1 × 10^5^ pfu/mouse) of HTNV strain 76–118 intramuscularly. After 3 days of infection, the mice were killed, and the major tissues, including the heart, liver, spleen, lung, kidney, and cerebrum, were individually collected for subsequent assays.

#### Detection of HTNV antigens in mouse tissues by ELISA

Each tissue sample was weighed, diluted in PBS, ground, and then freeze-thawed (−80/37°C) three times to prepare a 10% (g/mL) tissue suspension. The suspension was centrifuged at 12,000 rpm for 10 min at 4°C to collect the supernatant. HTNV antigens in the supernatant were detected by sandwich ELISA. The mAb 1A8 was used as a coating antibody, and HRP-conjugated 1A8 was used as the detecting antibody. Normal tissue supernatant was used as a negative control. The absorbance was measured at a wavelength of 450 nm using a Synergy HT ELISA plate reader (Biotec, Dresden, Germany). Absorbance of >0.1 was required as positive and positive/negative (P/N) >2.1 was considered significant.

#### HTNV nucleic acid detection in tissues by qRT-PCR

Total cellular RNA was extracted from each tissue sample using an RNAprep Pure Tissue Kit (Tiangen, Beijing, China), then 1 μg of total cellular RNA from each sample was used in a qRT-PCR using a SYBR Premix Ex Taq II Kit and the following HTNV RNA-specific primers: HTNV (forward) 5′-GATCAGTCACAGTCTAGTCA-3′ and HTNV (reverse) 5′-TGATTCTTCCACCATTTTGT-3′. GAPDH was included in the qRT-PCR as an internal control for cellular RNA using the following mouse GAPDH-specific primers: GAPDH (forward) 5′-AGGCCGGTGCTGAGTATG TC-3′ and GAPDH (reverse) 5′-TGCCTGCTTCACCACCTTCT-3′.

#### Histopathological analysis

Each tissue sample was embedded in a paraffin block and cut with a microtome into 5-μm sections, which were subsequently mounted onto glass slides and stained with hematoxylin and eosin (H&E).

### Flow cytometric analysis

Flow cytometric analysis was performed immediately with FlowJo version 9.2 (TreeStar). FITC-, PE-, and PerCP-Cy5.5-conjugated mouse IgG1, κ were used as isotype controls of the 3-color staining method. Lymphocytes were defined by FSC/SSC, and CD8^+^ T cells were defined as CD3^+^CD8^+^ events, which were then displayed on flow cytometric plot. The cytokine response was considered positive when the percentage of cells producing the cytokine was greater than 0.1% after background subtraction.

### Statistical analysis

Data were analyzed for statistical significance using GraphPad Prism 5 software. All data are expressed as the mean ± the standard error of mean (SEM) and are representative of at least three independent experiments. Comparisons of positive rate were analyzed by one way ANOVA. Results of ELISPOT assay were compared by one way ANOVA, and CTL assay was compared using two way ANOVA. Protective efficacy was compared using one way ANOVA. A *p*-value of 0.05 or less was considered significant.

## Results

### Identification of novel H-2K^b^-restricted CTL epitopes within the glycoprotein of HTNV by ELISPOT assay

**Figure 1:**
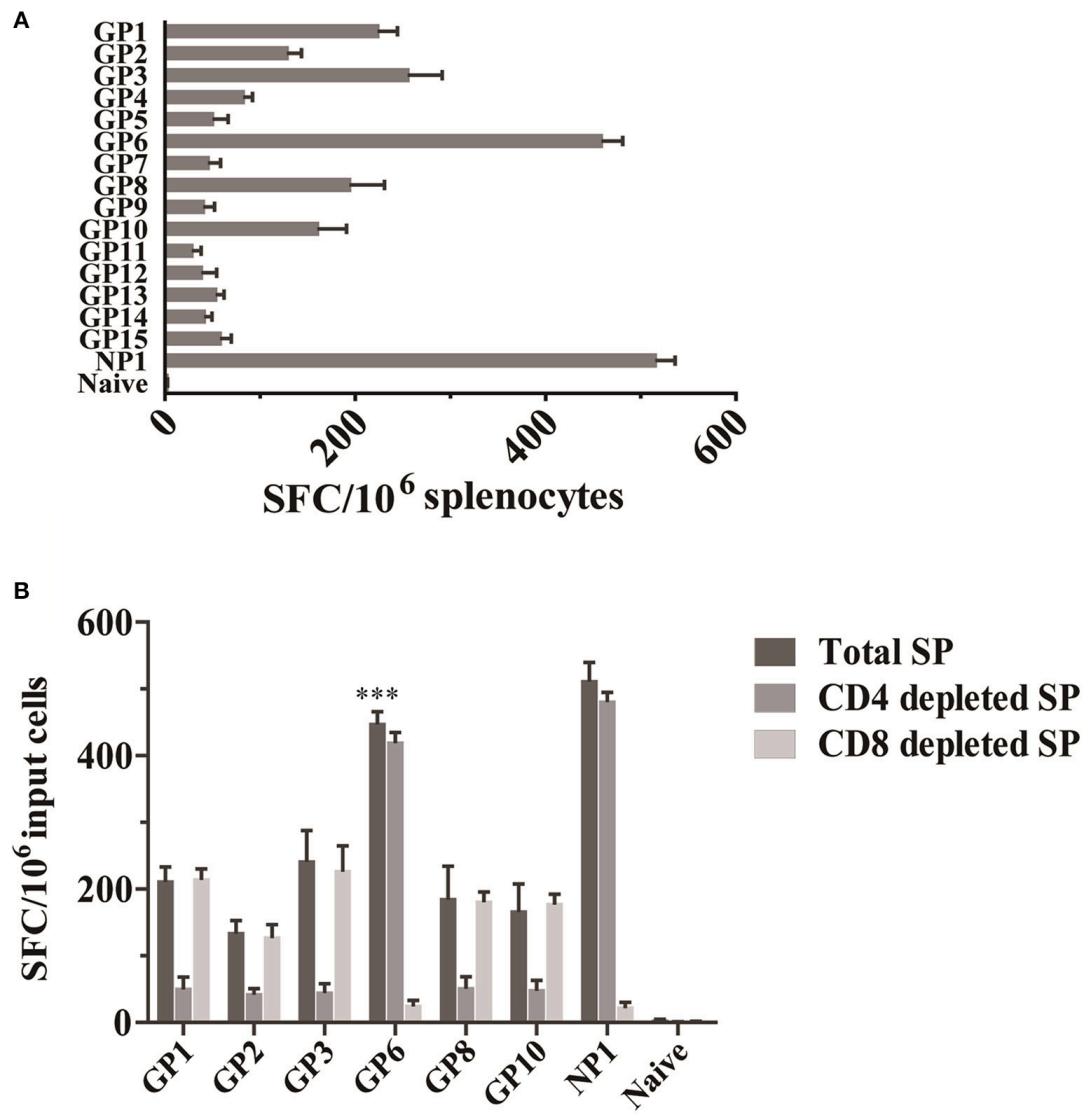
**Identification of an HTNV GP-specific CD8^**+**^ T cell epitope by IFN-γ ELISPOT assay. (A)** IFN-γ ELISPOT analysis of splenocytes obtained 7 d after HTNV infection (*n* = 6). Cells were stimulated with 15 8-mer peptides corresponding to HTNV glycoprotein. **(B)** IFN-γ ELISPOT analysis of splenocytes obtained 7 d after infection (*n* = 6) with the depletion of CD4^+^ T cells (CD4 depleted SP) and CD8^+^ T cells (CD8 depleted SP) or without depletion (Total SP). ^***^P < 0.0001.

To examine the T cell responses specific for HTNV-glycoprotein in C57BL/6 mice, we first screened T cell epitopes on HTNV-glycoprotein. Splenocytes isolated from HTNV-infected mice were tested against each peptide by enumeration of peptide-specific IFN-γ SFC using an *ex vivo* ELISPOT assay. Peptides GP1, GP2, GP3, GP6, GP8, and GP10 were found to induce IFN-γ production with a spot magnitude of 225, 130, 257, 461, 196, and 162 SFC/10^6^ input cells, respectively, whereas the remaining peptides only elicited a weak response (Figure 1A). Notably, the GP6 peptide elicited the strongest IFN-γ response among the various peptides tested, with a frequency of 461 SFC/10^6^ input cells, which was similar to the 516 SFC/10^6^ input cells of NP1. This finding indicates that six of 15 HTNV GP-specific peptides could induce a strong IFN-γ response.

Then, to determine a more precise understanding of the epitope-specific T cell responses, CD4^+^ or CD8^+^ T cell-depleted splenocytes from HTNV-infected mice were employed as effector cells. The results showed that one peptide (GP6 aa456~463: ITSLFSLL) induced significant numbers of IFN-γ-producing cells in CD4^+^ T cell-depleted cultures and limited the response magnitude in CD8^+^ T cell-depleted cultures. GP6 peptide induced a significantly higher numbers of IFN-γ spots in total splenocytes cultures compared to the spots in CD8^+^ T cell-depleted cultures (*p* < 0.0001), but significant difference has not been observed when compared to the spots in CD4^+^ T cell-depleted cultures. This finding confirms that GP6 is a CTL epitope on HTNV GP. However, CD4^+^ T cell depletion completely abrogated the IFN-γ responses induced by another 5 peptides (GP1, GP2, GP3, GP8, and GP10; Figure 1B), indicating that these peptides are CD4^+^ T cell epitopes that could be immunodominant.

### The cytotoxic capacity of HTNV glycoprotein-specific CD8^+^ T cells

**Figure 2:**
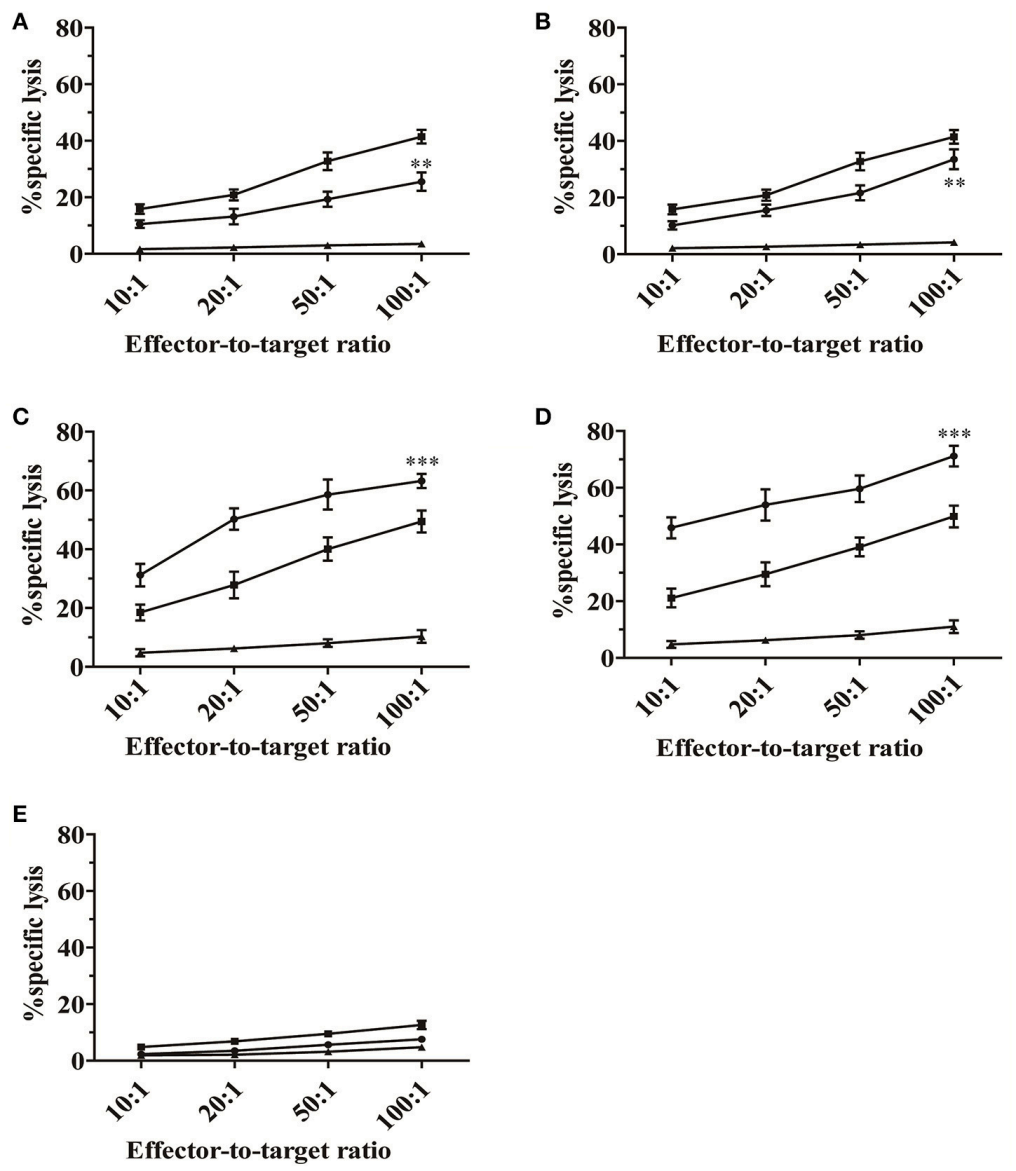
**Cytotoxic activity of splenocytes derived from HTNV-infected mice ***in vitro*****. The primary antiviral CTL responses were first detected. Splenocytes from C57BL/6 mice infected with HTNV (1 × 10^5^ pfu/head) were used as effector cells. As targets, EL-4 cells were pulsed with GP6 **(A)** and NP1 **(B)** at a concentration of 10 μg/mL. Splenocytes that were restimulated with GP6 **(C)** and NP1 **(D)** were used as effector cells in the second round of the CTL assay. Macrophages infected with HTNV (■) and peptide-pulsed EL-4 cells (•) were used as target cells. P815 cells (▴) were used as negative controls. **(E)** Evaluation of the mean lysis percentage of naïve CD8+T cells to kill no peptide-pulsed target cells. Macrophages (■), EL-4 cells (•), and P815 cells (▴) were served as target cells, respectively. Data are expressed as the mean ± SEM (*n* = 6). ^**^P < 0.001, ^***^P < 0.0001.

To further characterize the GP6 peptide as a CTL epitope, cytotoxic assays using GP6 and NP1 were performed. As described in methods, HTNV-infected macrophages, peptide-pulsed EL-4 cells and P815 cells (control cells) were used as target cells. As shown in Figure 2A, EL-4 cells pulsed with GP6 were killed by primary antiviral CTLs, with cell death percentages of 25.5, 19.3, 13.2, and 10.5% at E/T ratios of 100:1, 50:1, 20:1, and 10:1, respectively. The NP1 peptide sensitized target cells for lysis by anti-HTNV CTLs, with percentages of 33.5, 21.7, 15.5, and 10.2% at E/T ratios of 100:1, 50:1, 20:1, and 10:1, respectively (Figure 2B). As endogenous target cells, the HTNV-infected macrophages were also lysed by the primary antiviral CTLs at higher rates of 41.4, 32.7, 20.8, and 15.8%, at each E/T ratio mentioned above, respectively. These results indicate that GP6 can elicit CTL responses and plays a role in the primary antiviral response of HTNV-infected mice.

A second cell-mediated cytotoxicity assay was performed to evaluate whether GP6 could enhance peptide-specific cytotoxicity of CTLs. Splenocytes from HTNV-infected mice were restimulated *in vitro* with GP6 and NP1. Figure 2C shows that restimulation with GP6 resulted in strong CTL responses with percentages of cell death of 63.2, 58.6, 50.3, and 31.2% at E/T ratios of 100:1, 50:1, 20:1, and 10:1, respectively. NP1 peptide-specific cytotoxicity was greater with higher percentages of 71.1, 59.6, 53.9, and 45.9%, respectively (Figure 2D). Although, GP6 induced a relatively weaker CTL response compared to NP1, this peptide caused strong cytotoxic activity, which was especially significant at the E/T ratios of 50:1 and 20:1. Significant CTL activity could not be found in the non-specific group (Figure 2E). Other peptides were also tested, but CTL activity was undetectable. Collectively, these results suggest that the GP6 peptide is able to provide restimulation for primary antiviral CTLs to maintain specific cytotoxicity ability.

### Effector functions of HTNV GP epitope-specific CD8^+^T cells

**Figure 3:**
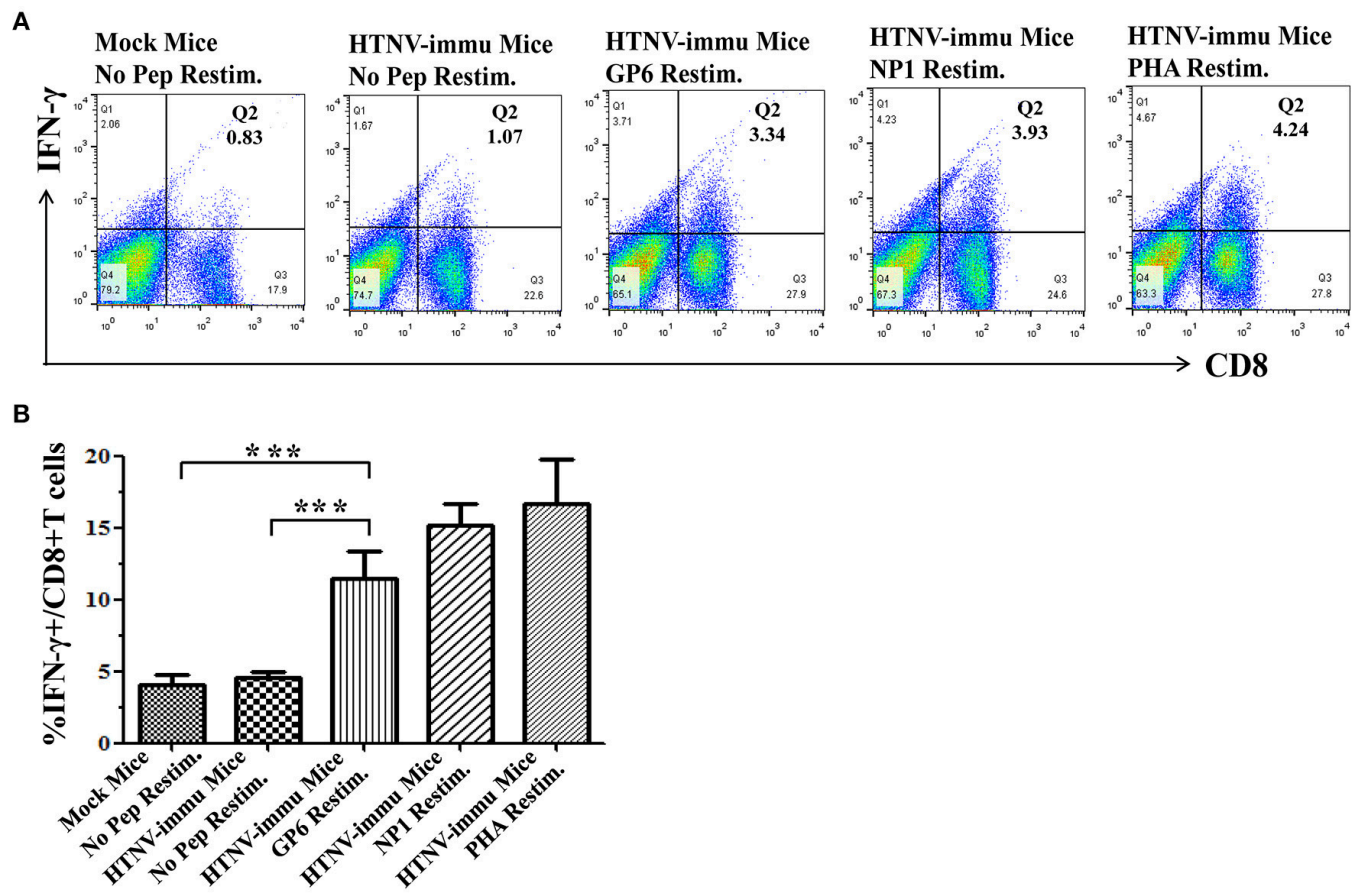
**The rapid secretion of IFN-γ after recognition of the HTNV-specific peptide. (A)** Representative flow cytometric plots of IFN-γ production by peptide-specific CD8^+^ T cells. The upper lane **(A)** shows the results 4 h after peptide restimulation and both negative and positive controls, and the lower lane **(B)** shows the frequency of IFN-γ production in CD8^+^ T cell populations. Data are expressed as the mean ± SEM (*n* = 6). ^***^P < 0.0001.

Given the importance of IFN-γ production in the defense against HTNV infection, we further investigated the production of IFN-γ by CD8^+^ T cells specific for HTNV GP by intracellular cytokine staining. This assay showed that the GP6 peptide induced a high percentage of IFN-γ production among CD8+ T cells (11.97% of total CD8^+^T cells). The frequency of IFN-γ-producing cells upon recognition of NP1 peptide was also observed (15.98% of total CD8^+^T cells). CD8^+^T cells restimulated with PHA also showed a high level of IFN-γ secretion with 15.25 percent of the total. However, a few cells producing IFN-γ were detected in both negative control groups (4.64% of total CD8^+^T cells in mock-infected mice with no peptide restimulation and 4.73% of total CD8^+^T cells in HTNV-infected mice with no peptide restimulation; Figure 3A). This assay was performed and repeated six times. We have given the statistical results in Figure 3B. HTNV-infected mice with GP6 restimulation group induced a significantly IFN-γ response compared to both negative control groups (the mock-infected mice with no peptide restimulation, *p* < 0.0001; HTNV-infected mice with no peptide restimulation, *p* < 0.0001). Our result is consistent with the data obtained in the ELISPOT assay (Figure 1).

### *Ex vivo* CD8^+^ T cell expansion against HTNV glycoprotein

**Figure 4:**
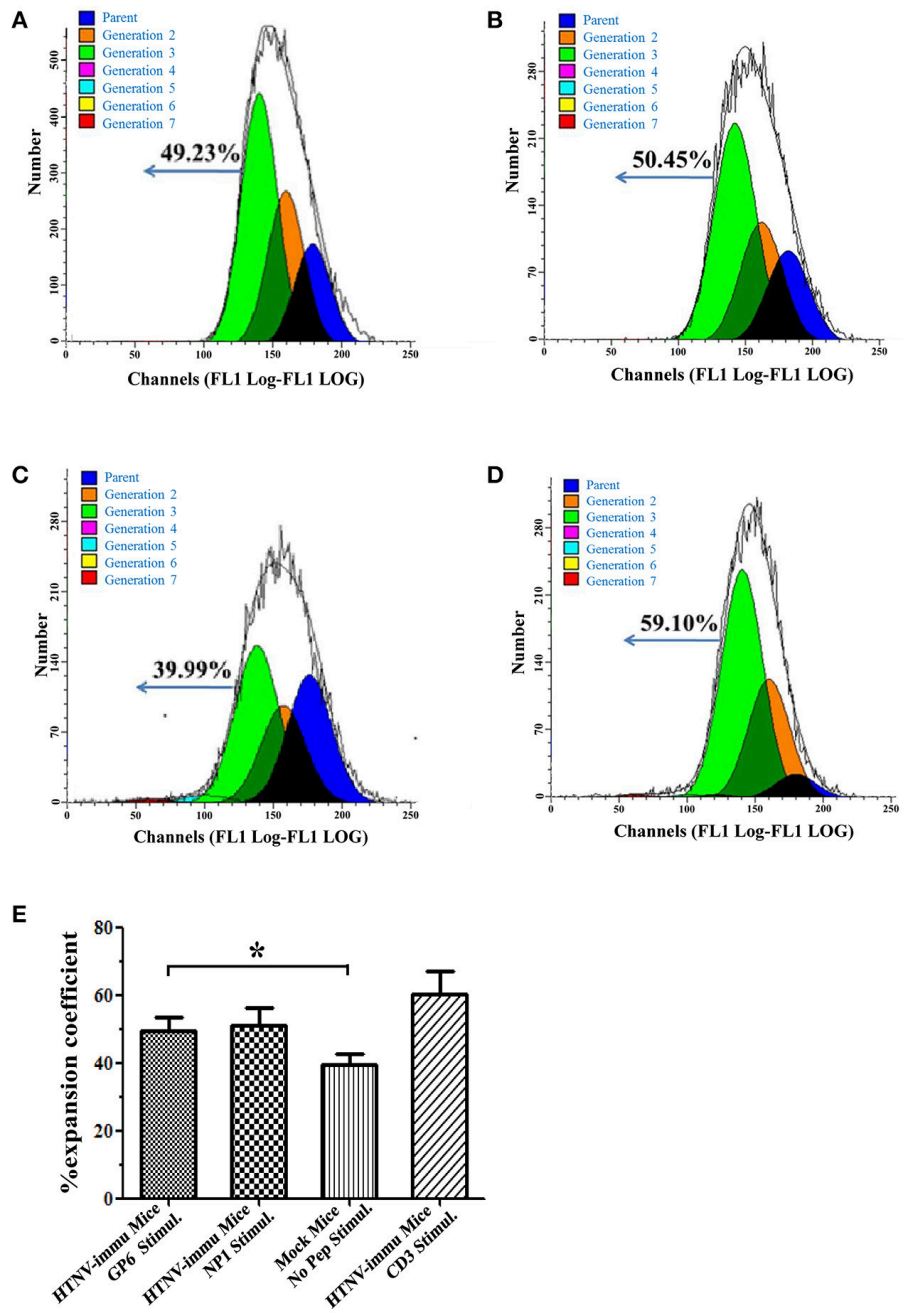
**The proliferative capacity of HTNV peptide-specific CD8^**+**^ T cells**. Representative flow cytometric histogram of the expansion percentage of CD8^+^ T cells stimulated by the GP6 **(A)** and NP1 **(B)**. Anti-mouse CD3 (Biolegend) **(D)** stimulation or no peptide **(C)** stimulation served as positive or negative controls, respectively. **(E)** The statistical data of the proliferation assay. Data are expressed as the mean percentage ± SEM (*n* = 6). ^*^P < 0.05.

We next assessed whether the antiviral effect of CTLs was dependent on the capacity of expansion. The CFSE staining assay showed that expansion of HTNV peptide-specific CTLs was readily detectable. As shown in Figure 4, PBMCs of HTNV-infected mice after stimulation with GP6 had a considerable expansion potential with 49.23% cells belonging to Generation 3 (Figure 4A). A similar level of expansion potential was observed in the NP1 stimulation group (Figure 4B). However, when stimulated with anti-mouse CD3 as a control, PBMCs had proliferative activity with 59.1% of cells belonging to Generation 3 (Figure 4C), which was higher than cells stimulated with HTNV-specific epitopes. The negative control of no peptide stimulation showed a low expansion coefficient with 39.99% (Figure 4D). The statistical results were provided in Figure 4E. HTNV-immunized mice with GP6 stimulation group showed a significant proliferation ability compared to the group with no peptide stimulation (*P* < 0.05). Previous studies have demonstrated a strong linkage between HTNV-specific CD8+T cell expansion and the control of HTNV infection. These data demonstrate the proliferative potential and capacity of specific CD8+ T cells in protecting from HTNV infection.

### Immunization with HTNV CTL epitope peptides elicited an enhanced cellular immune response in mice

**Figure 5:**
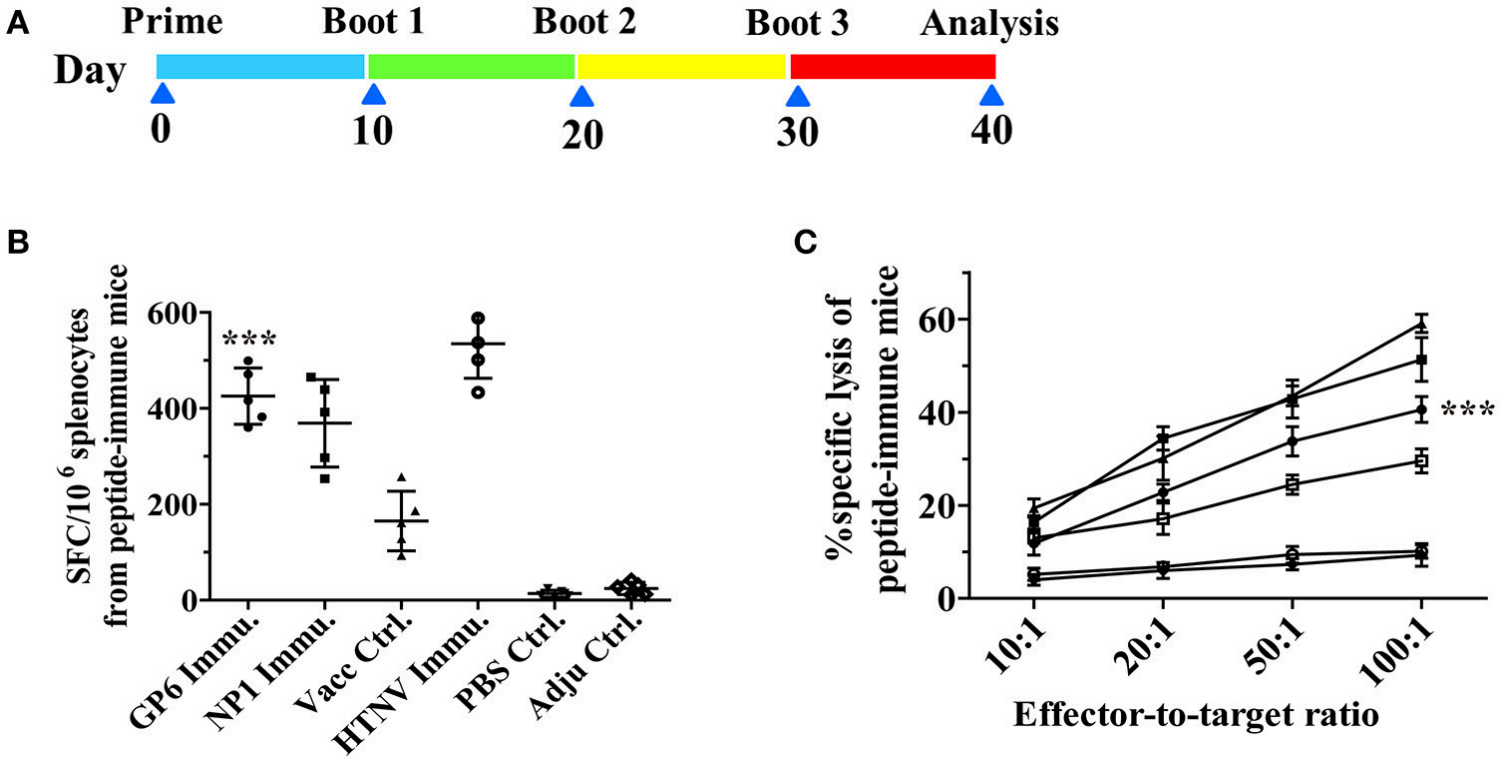
**The enhanced cellular immune responses in peptide-immune C57BL/6 and the timeline of immunization. (A)** Scheme of peptide immunization. Mice were primed and boosted with GP6 and NP1 packaged with Freud's adjuvants (Pep Immun group) or PBS (Mock group). Ten days after the final boost, splenocytes from mice were harvested for subsequent assays. **(B)** IFN-γ ELISPOT analysis of splenocytes obtained from HTNV peptide-immunized mice. Cells were stimulated with purified HTNV NP. **(C)** Splenocytes from immunized mice, restimulated with relative peptide were used as effector cells. As targets, EL-4 cells were pulsed with GP6 or NP1 at a concentration of 10 μg/ml. (■) represents NP1 immunization; (•) represents GP6 immunization; (▴) represents a positive control from the HTNV-challenged mice; (□) represents vaccine control; (◦) represents PBS control; (▾) represents adjuvant control. Data are expressed as the mean ± SEM (*n* = 6). ^***^P < 0.0001.

First, we measured cytokine production by ELISPOT assay as an indicator of cellular immune responses to reflect T cell responses to HTNV GP peptides. As shown in Figure 5B, splenocytes from GP6 peptide-immunized mice, restimulated with purified HTNV NP, presented a significantly higher number of IFN-γ spots compared to both the HTNV NP1-immunized group and inactivated Hantavirus vaccine group. HTNV-immunized mice showed the highest number. In contrast, we also observed that the numbers of IFN-γ spots from the two negative control groups (resolver control and adjuvant control) were extremely low.

Next, the GP peptide-specific CTL activity was detected by an LDH release assay. The cytotoxicity of splenocytes from mice immunized with GP6 was enhanced in accordance with the E/T ratio, which was the most significant at the ratio of 100:1 (Figure 5C). Splenocytes from the NP1-immunized mice showed the highest specific cytotoxic activity at E/T ratios of 100:1, 50:1, and 20:1 compared to those of the other groups. Additionally, the specific cytotoxic activities were not significant in the vaccine group. Perhaps, poor immunogenicity of these vaccines to elicit cell-mediated immunity is the main cause. However, non-specific cytotoxicity in the two negative control groups was extremely weak at E/T ratios of 100:1, 50:1, and 20:1.

Based on these results, immunization with GP6 peptide induced a high level of IFN-γ response and strongly specific cytotoxic activity, suggesting that immunization with HTNV-GP specific peptides enhances cellular immune responses.

### Immunization with recombinant CTL epitope peptides induced protective immunity in C57BL/6 mice against HTNV challenge

**Figure 6:**
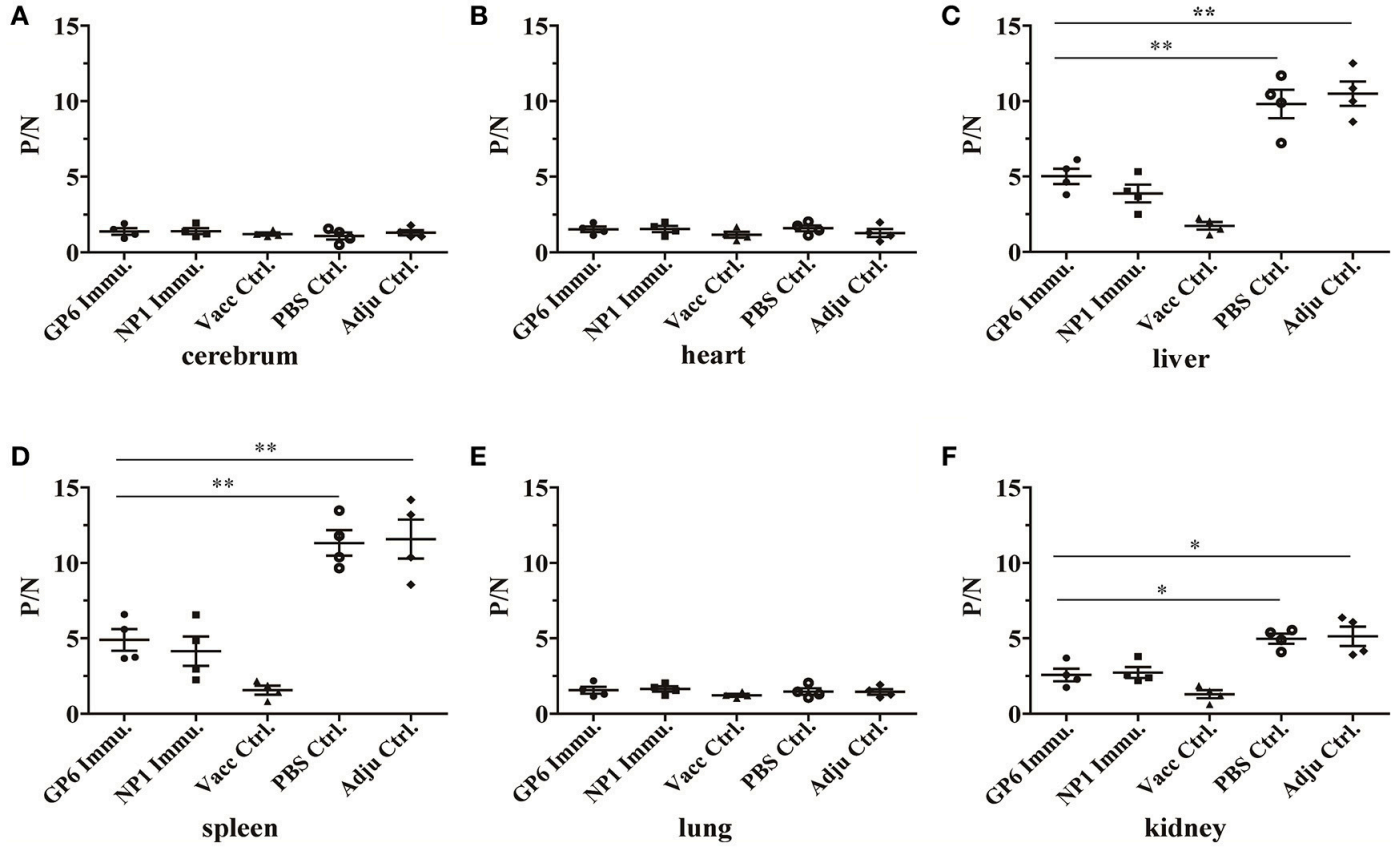
**Detection of HTNV-specific antigens from peptide-immunized mice by ELISA**. ELISA was used to detect HTNV antigens in the tissues of each group including the cerebrum **(A)**, heart **(B)**, liver **(C)**, spleen **(D)**, lung **(E)**, and kidney **(F)**. (•) represents the GP6 peptide-immunized group; (■) represents the NP1 peptide-immunized group; (▴) represents the inactivated Hantavirus vaccine control group; (◦) represents the PBS control group; (♦) represents the adjuvant control group. Data are expressed as the mean ± SEM (*n* = 4). ^*^P < 0.05, ^**^P < 0.001.

Protective immunity was evaluated in peptide-immunized mice. Three days after infection with HTNV, the major tissues of the mice were isolated to test against HTNV antigens by ELISA. We found that HTNV antigens were detected in the liver, spleen, and kidney of C57BL/6 mice in both negative control groups (adjuvant control and PBS control; P/N = 10.49, 11.57, and 5.13 in adjuvant control group or 9.89, 11.3, and 4.89 in PBS control group, respectively). However, antigens could not be detected in the liver, spleen or kidney of mice in the inactivated Hantavirus vaccine control group. Interestingly, this method detected lesser amounts of HTNV antigens in the liver, spleen and kidney of GP6-immunized mice compared with negative control groups that had absolutely no protection (P/N = 5.01, 4.90, and 2.57, respectively). Additionally, the HTNV antigens in NP1 peptide-immunized mice were found at low values of 3.67, 4.15, and 2.74, respectively. HTNV-specific antigens could not be detected in the heart, lung, or cerebrum of C57BL/6 mice from all of the groups (Figure 6). These results indicate that the immunization of C57BL/6 mice with the recombinant positive peptides provides protective immunity against HTNV challenge.

**Figure 7:**
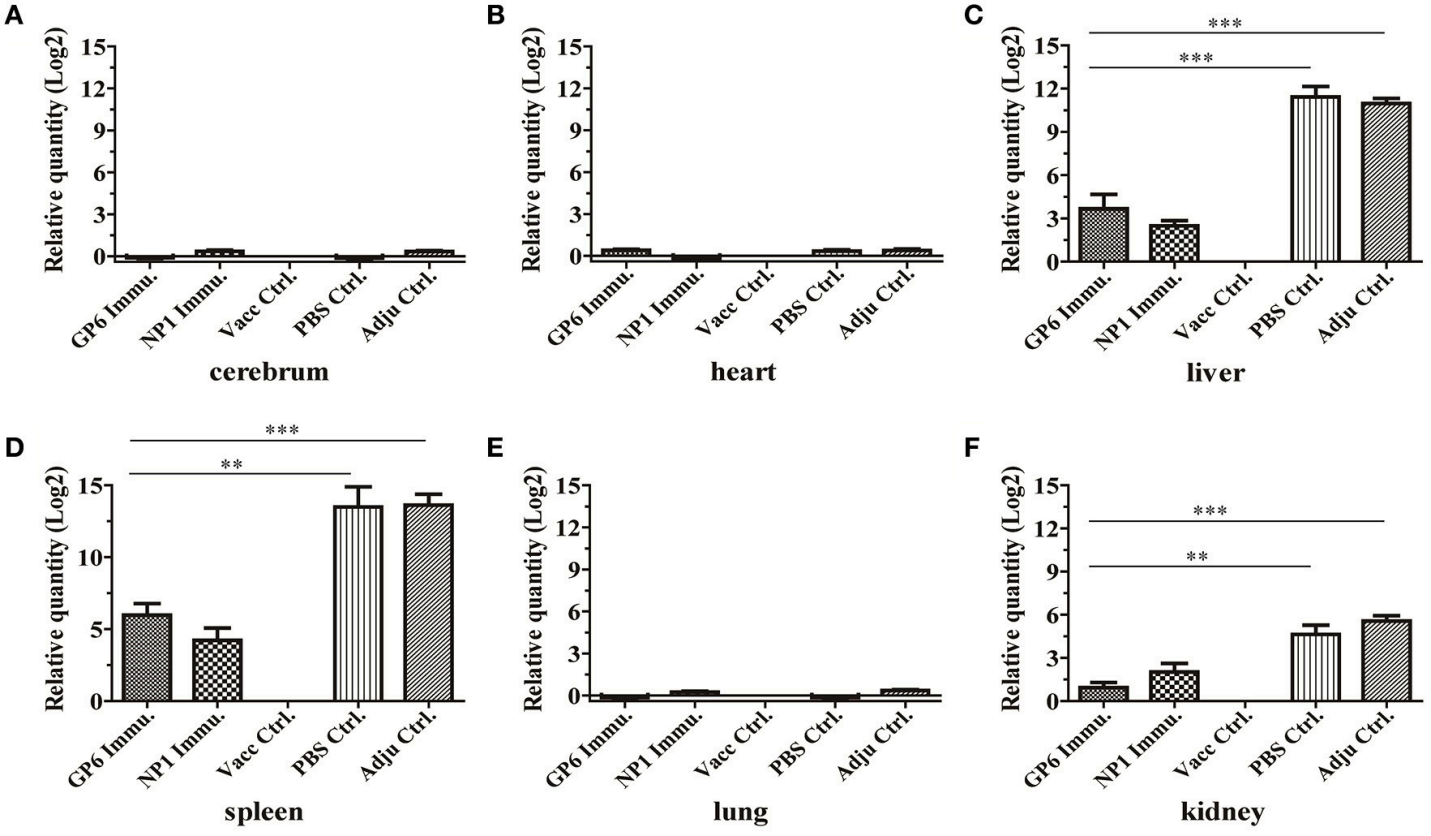
**qRT-PCR results of HTNV-specific nucleic acids in organs of peptide-immunized mice**. This method detected HTNV nucleic acid in the tissues of each group including the cerebrum **(A)**, heart **(B)**, liver **(C)**, spleen **(D)**, lung **(E)**, and kidney **(F)**. The viral nucleic acids are distributed mainly in the spleen and liver, but to a lesser degree in the kidney. Data are expressed as the mean ± SEM (*n* = 6). ^**^P < 0.001, ^***^P < 0.0001.

Meanwhile, the qRT-PCR assay was used to detect HTNV nucleic acids in the tissues of peptide-immunized C57BL/6 mice after challenge with HTNV. HTNV nucleic acids were detected in the liver, spleen, and kidney of C57BL/6 mice in the two negative groups with 11.43-, 13.5,- and 4.63-fold or 10.96-, 13.6,- and 5.57-fold amplification, respectively, compared to the inactivated Hantavirus vaccine group which did not contain viral nucleic acids. Small amounts of HTNV-specific nucleic acids were detected in the same tissues of C57BL/6 mice from the GP6- or NP1-immunized groups, compared to the negative controls (Figures 7C,D,F). Moreover, HTNV-specific nucleic acids could not be detected in other tissues of C57BL/6 mice in all of the groups (Figures 7A,B,E). These results were consistent with the ELISA results.

**Figure 8:**
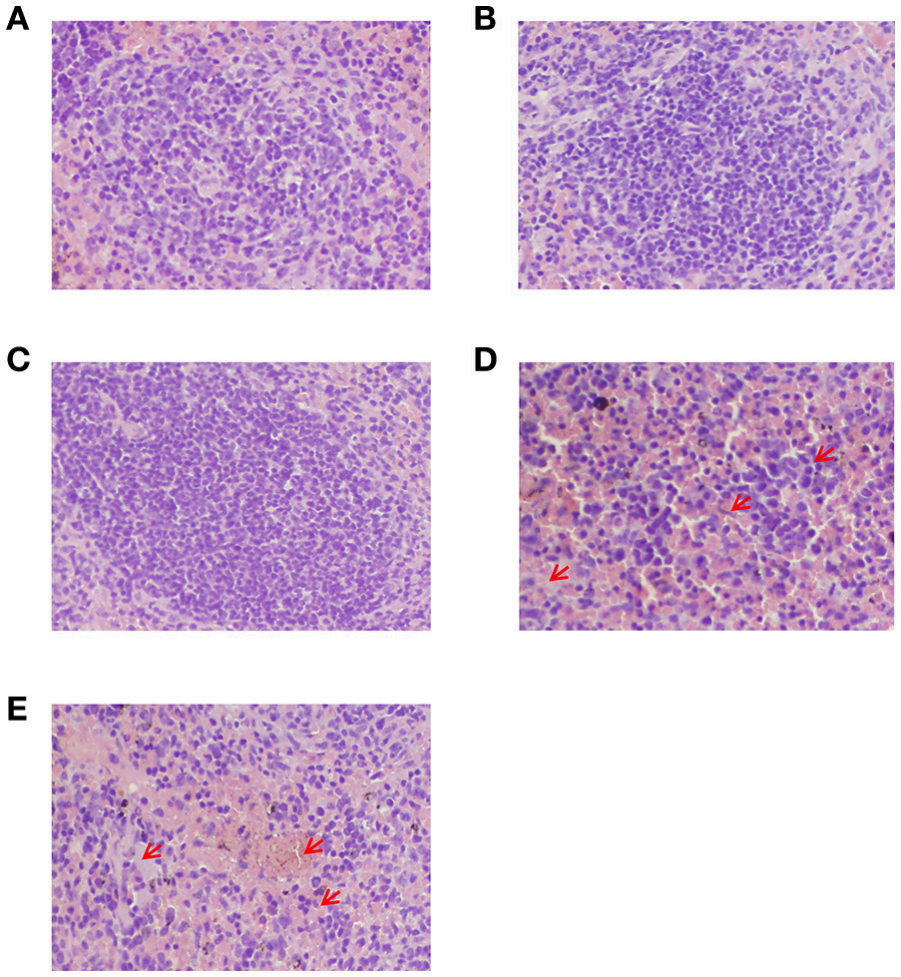
**Histopathological analysis of the spleens from peptide-immunized mice after challenge with HTNV by H&E staining. (A)** The GP6 peptide-immunized group. **(B)** The NP1 peptide-immunized group. **(C)** The inactivated Hantavirus vaccine group. **(D)** The PBS control group. **(E)** The adjuvant control. Red arrows show diffused lymphocyte infiltration, diffused hemorrhaging and increased white pulp in the spleens of two control groups.

Histopathological analysis by H&E staining showed that no significant alterations were found in the tissues from all of the groups except for the spleens of the two negative control groups (PBS control and adjuvant control). The results indicated some pathological changes including diffused lymphocyte infiltration, diffused hemorrhaging and increased white pulp in the spleens of the two negative control groups (PBS control and adjuvant control) which were marked with red arrows in the Figures 8D,E; however, these changes were not found in the other groups (Figure 8).

Taken together, these results show that immunization with the recombinant positive peptides induces protective immunity in C57BL/6 mice against HTNV challenge.

## Discussion

Vaccines, collectively a successful strategy for the prevention of infectious diseases, can direct the host's immune system against pathogens (Ma et al., 2016). Inactivated vaccines for HTNV have been shown to boost the humoral immune response (Kumar et al., 1997), but poor immunogenicity in eliciting neutralizing antibodies and limited cell-mediated immunity following vaccination, as well as concern regarding safety are major obstacles for development (Yang et al., 2005). Therefore, inactivated vaccines are not expected to be able to effectively inhibit HTNV amplification to completely prevent viral infection (Yang et al., 2005). A potent T cell-activating peptide vaccine based on the HTNV structural protein may be a promising strategy for disease control (Rubsamen et al., 2014; Lu et al., 2016). The T cell response is a critical element of naturally acquired immunity to control viral infection, including HTNV infection (Rasmuson et al., 2011). This fact provided the theoretical basis for the development of HTNV peptide vaccines to fight infection by inducing specific T cell responses. In this study, we reported that the GP6 peptide derived from glycoprotein of HTNV could be an immunogenic epitope based on its ability to induce peptide-specific CD8+ T cell responses and on *in vivo* functional validation. We further provided evidence that immunization with GP6 in C57BL/6 mice successfully induced T cell responses with high production of IFN-γ and potent cytotoxicity, which involved in combatting HTNV infection after HTNV challenge. This reveals that GP6 peptide may be a good candidate for use as a peptide-based vaccine in the design of preclinical studies *in vivo*.

### Identification of the correct CTL epitopes within the glycoprotein of HTNV by ELISPOT assay

Selecting the correct epitope is a priority when designing an effective vaccine. An epitope, which is the portion of an antigen presented on major histocompatibility complex (MHC) molecules that bind the TCR or BCR of T/B lymphocytes, initiates, and evokes T/B cell immune responses (Hoffmeister, 2003). First, we evaluated the sequence of the HTNV GP using an algorithm, provided by IEDB of the National Institute of Allergy and Infectious Diseases (Vita et al., 2015). We selected and synthesized 15 peptides and tested their respective antigenicity according to binding score. A higher score means a stronger bind to MHC (Park et al., 2000). This first crucial step increases the probability of successful epitope selection. The high production of antiviral cytokines is a critical characteristic of effective T cell immunity for both suppressing viral replication and eliminating virus-infected host cells. IFN-γ has been implicated in immune regulation and direct antiviral activities (Frese et al., 2002; Gattoni et al., 2006). Therefore, the IFN-γ ELISPOT was conducted for mapping the epitopes of HTNV glycoprotein. In the first round of the ELISPOT assay, six of 15 peptides could induce a strong IFN-γ response, but this datum does not illustrate that the peptides are CTL epitopes. Taking the complexity of IFN-γ-secreting cells into consideration, the second round of the ELISPOT assay was performed using both CD4^+^ T cell-depleted splenocytes and CD8^+^ T cell-depleted splenocytes as effector cells. We tried to use CD4^+^ T cell-depleted splenocytes as effector cells to recognize the CD8^+^T cell responsive 8-mer epitopes. On the other hand, CD8^+^ T cell depletion completely abrogated the IFN-γ response indicating that these peptides were CD8^+^ T cell epitopes. The data showed that HTNV GP6 induced the largest number of IFN-γ-producing cells (over 450 cells per 10^6^ splenocytes) with CD4 depletion and a small quantity with CD8 depletion (less than 40 cells per 10^6^ splenocytes) among the 15 alternative peptides. These data are consistent with that of NP1, which also induced a significant number of IFN-γ-producing cells (over 470 cells per 10^6^ splenocytes) when CD4^+^T cells were depleted and <35 SFC when CD8 cells were depleted (Figure 1B). However, other peptides did not induce detectable levels of IFN-γ-producing cells when the CD4^+^ T cells were depleted. Based on this finding, we speculate that GP6 might be an immunodominant CTL epitope.

### Detection of the cytotoxicity of HTNV epitope-specific CD8^+^ T cells by CTL assays

CTL assays were performed to evaluate peptide-specific cytotoxic activity. The data suggested that GP6 elicits a CTL response and also plays a key role in the primary antiviral response during HTNV infection in mice. To examine whether this peptide could enhance peptide-specific cytotoxicity of CTLs, we measured secondary CTL responses after *in vitro* restimulation. After secondary stimulation with the GP6 peptide, CTL activity against the relevant peptide was increased. At the same time, we also used HTNV-infected macrophages as the endogenous target cells to evaluate whether the peptide-specific CTLs could effectively recognize endogenously processed antigen. The results showed a high level of cytotoxic activity of GP6-specific CTLs even when using the endogenous cells as targets. This suggests that GP6 could be processed and presented by endogenously antigen-presenting system. These results also imply that GP6 can provide restimulation to maintain the cytotoxic activity of primary antiviral CTLs and have the potential to induce T lymphocyte responses during viral infection.

### CTL epitope from HTNV glycoprotein provided significantly enhanced immunogenicity over inactivated HTNV vaccine

To evaluate the immunogenicity of the identified epitopes from our study *ex vivo*, the cellular immune response was evaluated at 10 d after the final booster immunization with the specific epitope in C57BL/6 mice. Our results showed that the splenocytes of GP6 group mice always showed stronger T cell responses compared with the other groups. Meanwhile, the splenocytes of the vaccine control group have a weak magnitude of response of T cells, particularly because of the poor immunogenicity of inactivated Hantavirus vaccine to elicit cell-mediated immunity (Kwilas et al., 2014). Our results verified this point again.

### The CTL epitope-vaccinated mice presented partial protection immunity from challenge with HTNV

We further provided evidence of the potential capacity of the identified epitopes to protect mice after HTNV challenge through the animal protection assay. Viral load in each of the major organs of C57BL/6 mice served as parameters to assess protection. Based upon the peak of viral replication, the mice were sacrificed 3 days post-challenge. Using ELISA and qRT-PCR, we found that large amounts of HTNV antigens and nucleic acid could be detected in the spleen, liver, and kidney of C57BL/6 mice in the two negative control groups (PBS control and adjuvant control), but not in the vaccine group. Further comparisons revealed that viral antigens and nucleic acids in the spleen were higher than those in the liver and kidney. It was interesting to note that viral antigens and nucleic acids can only be detected in small amounts in the GP6 and NP1 groups. The data from histopathological analysis by H&E staining also showed some pathological changes in the spleens (Figure 8) of mice belonging to the two negative control groups (PBS control and adjuvant control) but not in vaccine groups after challenge with HTNV. Although, the level of HTNV nucleic acids and antigens in the GP6 group was greater than those in the vaccine group, the potential capacity of GP6, as an 8-mer aa peptide existing on the huge HTNV structure, to protect mice from HTNV infection cannot be ignored. These findings suggests that GP6-specific CTL response could protect animals from HTNV challenge.

### An excellent method to evaluate protective therapeutic agent for HTNV

Our goal was to identify CTL epitopes on HTNV GP in mice to gain a better understanding of the recognition of HTNV by cellular immune responses, and we found that GP6 could be applied to vaccine design preclinical studies of vaccines that target T cell immunity. HTNV infection in adult mice results in transient viral replication with no symptoms followed by the elimination of virus (Araki et al., 2004). However, HFRS is a severe, life-threatening illness characterized by fever, hemorrhage, and renal failure (Serikawa and Yamada, 1993). Due to limitations of using a model for disease progression, suitable animal models that mimic HFRS are presently unavailable. Our research is based on detecting the viral antigens and nucleic acid to evaluate the protective ability of viral epitopes, and this method can also be used to evaluate each protective therapeutic agent for HTNV.

In summary, one novel CTL epitope peptide GP6 aa456-aa463 (ITSLFSLL) was identified in this study. As far as we know, this is the first report of a CTL epitope on HTNV glycoprotein in mice and we confirmed that the HTNV-specific GP6 peptide is a highly immunogenic epitope restricted by H-2K^b^. By challenging C57BL/6 mice with HTNV *in vivo*, we revealed the immunogenicity of vaccination with the HTNV GP6 peptide and the antiviral efficiency of GP6-specific CTL responses. But our present work only find the CTL epitope on HTNV GP, we should learn more about other epitopes on HTNV GP and NP (such as neutralizing epitope) and the immunological characteristics of their combined application in the next step. Our findings could make some contributions to the establishment of HFRS animal model and research on HFRS multiple peptides vaccine.

## Author contributions

RM and LC are responsible for the contents of main experiments and writing the manuscript. QY and RL are responsible for the flow cytometric analysis. TM, XZ, and ZL are responsible for the animal raising and immunization. LZ and WY are responsible for data collection. FZ and ZX are responsible for experiment instruction. XW and FW are responsible for experimental design. All authors read and approved the final manuscript.

### Conflict of interest statement

The authors declare that the research was conducted in the absence of any commercial or financial relationships that could be construed as a potential conflict of interest.
